# Hahnemann’s Essay

**Published:** 1889-03

**Authors:** 


### HAHNEMANN'S ESSAY.

Any words of advice upon the practice of medicine which come to us from the pen of Hahnemann must always be welcome to the lovers of truth. We, therefore, feel sure our readers will be thankful to Dr. F. H. Lutze for his translation of the essay upon "the Repetition of the Homœopathic Remedy," which we believe now appears in English for the first time. Dr. Lutze has given the German idea almost literally, thereby risking no chance of altering in any way the true meaning of the master's words. Dr. Lutze writes as follows:

"This essay was published by Dr. C. v. Bœnninghausen, as a preface from Samuel Hahnemann, to his (Dr. v. B.'s) repertory of *Antipsoric Remedies*, 2d edition, Münster, 1833. As very plainly appears from the accompanying extract (page 119) from Dr. C. v. Bœnninghausen's own preface to the same repertory, this essay was written and sent by Samuel Hahnemann himself to Dr. C. v. Bœnninghausen, who was anxious to give his brother homœopaths the benefit of it. I am not aware that it has ever appeared in the English language before, therefore, I have translated it, and offer the same through THE HOMŒOPATHIC PHYSICIAN to the English speaking part of the homœopathic profession."

